# Outcomes within 100 days of hematopoietic cell transplantation in pediatric patients: insights from an intensive care unit in Colombia

**DOI:** 10.3389/fped.2024.1497675

**Published:** 2024-11-15

**Authors:** Rubén E. Lasso-Palomino, Diego Medina, Alexis Antonio Franco, María José Soto-Aparicio, Eliana Manzi Tarapues, Diana Marcela Muñoz, Edgar Salazar, Jhon López, Angela Devia, Sofía Martínez-Betancur, Jimena Sierra, Anita V. Arias, Inés Elvira Gómez

**Affiliations:** ^1^ Fundación Valle del Lili, Unidad de Cuidado Intensivo Pediátrico, Unidad Materno infantil, Cali, Colombia; ^2^ Departamento de Pediatría, Facultad de Medicina, Universidad Icesi, Cali, Colombia; ^3^ Fundación Valle del Lili, Centro de Investigaciones Clínicas (CIC), Cali, Colombia; ^4^ Departamento de Pediatría, Servicio de Hematología, Fundación Valle del Lili, Cali, Colombia; ^5^ Facultad de Ciencias de la Salud, Universidad Icesi, Cali, Colombia; ^6^ Departamento de Pediatría, Facultad de Ciencias de la Salud, Universidad Tecnológica de Pereira, Pereira, Colombia; ^7^ Division of Critical Care and Pulmonary Medicine, Department of Pediatrics, St Jude Children’s Research Hospital, Memphis, TN, United States

**Keywords:** hematopoietic cell transplantation, critical care outcomes, pediatrics, pediatric intensive care unit, mortality, resource-limited settings

## Abstract

**Introduction:**

Hematopoietic cell transplantation (HCT) has become an essential therapeutic modality for pediatric patients with malignant and non-malignant conditions. Despite its effectiveness, many patients experience post-transplant complications often leading into life-threatening conditions requiring specialized care in a Pediatric Intensive Care Unit (PICU). This study aims to describe clinical characteristics associated with mortality in pediatric HCT patients who needed PICU care within 100 days post-transplant in a resource-limited country.

**Methods:**

A retrospective cohort study was conducted involving pediatric HCT patients (<18 years old) admitted to our PICU from January 2012 to December 2021. Variables were characterized according to their nature, employing appropriate measures of central tendency and dispersion. The relationship between mortality and patient clinical characteristics was assessed using the Chi-square test or the Mann-Whitney U test, as applicable. A *p*-value of <0.05 was considered statistically significant. A Kaplan Meier survival curve was performed considering the days from HCT to death during PICU admission and a Cox regression analysis was conducted to analyze the association between PRISM III score and risk of death. Data analysis was executed utilizing the STATA SE v18 statistical software package. Of 316 HCTs, 69 patients required admission to the PICU. Haploidentical transplants from related donors were performed in 72.5% of these patients. The primary cause of PICU admission was infection, accounting for 68.1% (*n* = 47) of cases. Factors significantly associated with mortality included a PRISM III score > 20 (*p* < 0.002), mechanical ventilation (*p* < 0.007), renal replacement therapy (*p* < 0.002) and vasoactive support (*p* < 0.001). A total of 27 patients succumbed during their PICU stay. Kaplan Meier curve showed a survival rate of 51.6% at100-days post-transplant. A PRISM III score higher than 20 points was related with mortality (Hazard ratio 5.71 CI 95% 2.09-15.5).

**Discussion:**

This study examines critical factors associated with mortality in pediatric HCT recipients who required admission to our PICU within the first 100 days post-transplant. Our findings indicate that infectious complications, alongside the need for advanced cardiovascular, respiratory, and renal support are strongly correlated with mortality. These results underscore the importance of early risk factor identification and targeted interventions to optimize patient outcomes.

## 1 Introduction

Hematopoietic cell transplantation (HCT) has emerged as a vital therapeutic strategy, that significantly improves survival for pediatric patients with both malignant and non-malignant conditions (1–6). However, despite advances in the field of HCT, a considerable number of patients require admission to a pediatric intensive care unit (PICU) due to complications of the transplantation process itself, treatment protocols, and pre-existing comorbidities (7). The leading causes for PICU admission include severe infections, graft-vs.-host disease (GVHD), and graft failure, which can cause critical complication such as respiratory failure, electrolyte imbalances, renal insufficiency, and multi-organ dysfunction (8–11). Alarmingly, PICU mortality rates in this vulnerable population can range from 16.2% to 22.1% (11, 12) in the post-transplant period, with survival following a single PICU admission estimated at only 43% (13). For patients requiring intubation and mechanical ventilation (MV), mortality can reach 44.0%–60.4% (1, 9, 11, 14), and for those needing renal replacement therapy (RRT), mortality can reach 51.9% to 56.1% (15–17). A study by Zinter et al. analyzing data from the Virtual Pediatric Systems (VPS, LLC) and Blood and Marrow Transplant Research (CIBMTR) databases between 2009 and 2014 in the United States, reported a PICU mortality rate of 17.5% in pediatric patients after allogeneic HCT, with rates exceeding 50% in those requiring MV and RRT (9).

Unfortunately, the existing literature has primarily focused on adult HCT patients in high-income countries, resulting in a significant knowledge gap regarding the unique challenges faced by pediatric patients, particularly in low- and middle-income countries (LMICs). This study seeks to better understand these challenges by examining the clinical characteristics associated with early complications and mortality among pediatric HCT patients who are admitted to the PICU within the first 100 days post-transplantation. This research is conducted in a resource-limited setting that treat pediatric patients with complex diseases in southwestern Colombia, where only 8 institutions are equipped to perform pediatric HCTs.

## 2 Materials and methods

### 2.1 Study design and setting

We conducted a comprehensive retrospective cohort study focusing on pediatric patients younger than 18 years of age who underwent HCT for malignant or non-malignant indications between January 2012 and December 2021, and who were subsequently admitted to the PICU within the first 100 days post-transplant at Fundación Valle del Lili. The primary inclusion criteria was admission to the PICU within the first 100 days post-transplant. Data collection focused on PICU admissions occurring within this 100-day period. For cases in which the PICU stay extended beyond the 100th day post-transplant, the data were included in the analysis for the full duration of the PICU stay. However, we did not consider other clinical information of hospital admissions to general ward 100 days after HCT.

Fundación Valle del Lili is a general hospital, located in the southwestern region of Colombia, with approximately 25% to 30% of its services dedicated to pediatric care. Fundación Valle del Lili is a referral center for high-complexity pathologies, playing a key role in managing severe and specialized cases. Pediatric transplant patients are admitted to a dedicated transplant unit with 13 beds, and the hospital also has a 40-bed PICU that admits around 200 patients per month. This facility serves as a critical resource in a region where healthcare facilities often face therapeutic and resource constraints due to high patient volumes. According to the Global Burden of Disease Study 2019 Socio-Demographic Index, Colombia is categorized as a middle-income country (18).

We excluded patients who underwent HCT at other institutions and those admitted to the PICU solely for monitoring during high-risk medication administration. Data were collected on demographic variables, and clinical characteristics—(i.e., medical history, type of transplant, and reason for PICU admission),—as well as complications and support measures utilized in the PICU. Support measures included invasive and non-invasive ventilatory support. The latter encompassed support through continuous positive airway pressure (CPAP), bilevel positive airway pressure (BPAP), and high-flow nasal cannula (HFNC). Mortality was assessed for all patients admitted to the PICU within the 100 days post-transplant, including those patients who were admitted near to the end of this period and whose death occurred after this timeframe. Additionally, we noted any deaths that occurred outside the PICU and within the initial 100 days post-transplant; however, these cases were not included in the mortality analysis. Specifically, patients who were under palliative care were excluded from the mortality analysis due to their unique clinical circumstances. Data were extracted from the patients’ medical records and tabulated using a standardized form.

### 2.2 Data analysis

Descriptive statistics were used. To ensure robust analysis, variables were characterized based on their nature and distribution by using measures of central tendency and dispersion. The Shapiro-Wilk test was used to assess the statistical distribution of numerical variables. Variables exhibiting a normal distribution were summarized using mean and standard deviation, while non-normally distributed variables were described with median and interquartile range (IQR). Categorical variables were summarized in proportions and percentages. We analyzed the association between clinical characteristics and mortality using the Chi-square test for categorical variables and the Mann Whitney U rank sum test for continuous variables. A survival analysis with Kaplan Meier method was used considering the time from HCT to death during PICU admission. A Cox regression model was performed using the Pediatric Risk of Mortality-III (PRISM III) score. We established a *p*-value threshold of <0.05 as statistically significant. All data analyses were conducted using the STATA SE v18.0 statistical software package.

### 2.3 Ethical considerations

This study was conducted in accordance with ethical guidelines and with the regulatory approval from the Clinical Research Ethics Committee of Fundación Valle del Lili in May 2022.

## 3 Results

During the study period, January 2012 to December 2021, a total of 316 HCTs were performed in pediatric patients. Out of these, 69 patients (21.8%) met inclusion criteria. Notably, 13 of these 69 patients required a second admission to the PICU within the first 100 days post-transplant, and 5 required a third admission.

Table 1 outlines the demographic characteristics of our patients. The cohort included 49.3% male and 50.7% female patients, and a median age of 8 years (IQR 4 - 13). The majority of HCTs were conducted for malignant diseases (73.9%, *n* = 51), with acute lymphoblastic leukemia being the most prevalent condition (49.0%, *n* = 25). The predominant source of HCTs was haploidentical related donors (72.5%, *n* = 50), and peripheral blood was the most frequently utilized stem cell source (50.7%, *n* = 35).

**Table 1:** Demographic and HCT-related characteristics of pediatric patients admitted to the PICU within the first 100 days post-transplant (*n* = 69).

Characteristic	Total number	Percentage
Sex		
Male	34	49.3
Female	35	50.7
Age in years, median (IQR)	8 (4–13)	—
Indication for transplant		
Malignant disease/neoplasia	51	73.9
Bone marrow failure syndrome	8	11.6
Immunodeficiency	6	8.7
Hemoglobinopathies	3	4.4
Epidermolysis bullosa	1	1.5
Malignant pathology associated with transplant (*n* = 51)		
Acute lymphoblastic leukemia	25	49
Acute myeloid leukemia	10	19.6
Solid tumors	8	13.7
Non-hodgkin lymphoma	3	5.8
Myelodysplastic/myeloproliferative syndrome	3	5.8
Hodgkin lymphoma	2	3.9
Type of transplant		
Haploidentical related donor	50	72.5
Identical related donor	9	13.0
Autologous	8	11.6
Unrelated donor	2	2.9
Source of transplant cells		
Peripheral blood	35	50.7
Bone marrow	32	46.4
Umbilical cord blood	2	2.9

In terms of clinical characteristics during the PICU stay, infections were the leading cause of the first admission, representing 68.1% (*n* = 47) of cases. Bacteremia, defined as a bloodstream infection confirmed by the isolation of bacteria from at least one blood culture, was diagnosed in 49.3% (*n* = 34) of these patients. The most commonly isolated microorganism among patients with infections was bacteria (*n* = 34). Other significant causes for PICU admission included hemorrhagic events (10.1%, *n* = 7), neurological complications (7.25%, *n* = 5), and GVHD (5.80%, *n* = 4). The median length of stay of the first PICU admission was 5 (IQR 3-14) (see Table 2).

**Table 2:** Clinical characteristics and interventions of pediatric patients with HCT admitted to the PICU within the first 100 days post-transplant (*n* = 69).

Characteristic	Total number	Percentage
Cause of PICU admission		
Infection	47	68.1
Bleeding	7	10.1
Neurological disorder	5	7.2
Graft-vs.-host disease	4	5.8
Electrolyte imbalances	9	13.4
Days in PICU, median (IQR)	5 (3–14)	—
Days between transplant and PICU admission, median (IQR)	15 (5–41)	—
Infection focus (*n* = 47)		
Bacteremia	34	49.3
Genitourinary	5	7.2
Respiratory	5	7.2
Gastrointestinal	5	7.2
Central nervous system	2	2.9
Identified and isolated microorganisms (*n* = 39)		
Bacteria	34	47.8
Virus	7	10.1
Fungi	4	5.8
Identified microorganism (*n* = 45)		
Klebsiella pneumoniae	14	20.3
Pseudomonas aeruginosa	4	5.8
Escherichia coli	4	5.8
Staphylococcus epidermidis	2	2.9
Virus (rhinovirus, enterovirus, respiratory syncytial virus, polyomavirus, parainfluenza 1, cytomegalovirus)	6	10
Enterococcus faecalis	1	1.4
Pneumocystis jirovecii	1	1.4
Other	15	21.7
Patients with identified resistance pattern	24	55.8
Type of resistance pattern identified (*n* = 24)		
Carbapenemases	16	66.6
Extended spectrum beta-lactamases	6	25.0
Other	3	12.5
Complications		
Sepsis with septic shock	25	36.2
Ventilatory failure	11	15.9
Multiple organ failure	10	14.5
Neurological deterioration	8	11.6
Other	6	8.7
PRISM III score		
Score 0–12	32	46.4
Score 13–19	9	13
Score >20	28	40.5
Mortality related to transplant (*n* = 69)	27	39.1
In which admission did the patient died? (*n* = 28)		
First admission to PICU	21	75
Second admission to PICU	4	14.3
Third admission to PICU or afterwards	2	7.1
General ward	1	3.6
Cause of death during first admission (*n* = 21)		
Sepsis	14	66.6
Thrombotic microangiopathy	4	19
Diffuse alveolar hemorrhage	3	14.3
Graft-vs.-host disease	2	9.5
Hypoxemic ventilatory failure/PARDS	1	4.8
Veno-occlusive disease	1	4.8
Requirement for mechanical ventilation	41	59.4
Type of ventilation		
Non-invasive mechanical ventilation (NIV)	13	31.7
Invasive mechanical ventilation (IMV)	28	68.3
Requirement for vasoactive support	32	46.4
Requirement for renal replacement therapy	9	13
Did the patient require re-admission to the PICU?		
No	59	82.6
Yes	12	17.4
Days between first PICU admission and death, median (IQR)	15 (5–41)	—
Days between transplant and death, median (IQR)	48.5 (14.5–80.5)	—

Regarding interventions during the PICU stay, 59.4% (*n* = 41) of patients required ventilatory support, with 28 of them needing invasive mechanical ventilation (IMV). Additionally, 46.4% (*n* = 32) required vasoactive support, and 13.0% (*n* = 9) underwent renal replacement therapy. The Pediatric Risk of Mortality-III (PRISM III) scale indicated that 40.5% (*n* = 28) of the patients had a score greater than 20. During this admission, 21deaths occurred, with sepsis (66.6%) identified as the leading cause of mortality. One additional case of fatality happened in a patient outside the PICU, who was part of a palliative care program. The median time between the first PICU admission and death was 20.5 days (IQR 8.5-49), while the median time from transplant to death was 57 days (IQR 20.5-73.2).

Table 3 details the clinical characteristics and therapeutic interventions of 13 patients who required a second admission to the PICU during the study period. The median length of stay for this admission was 5 days (IQR 3-6), and the median time from transplant to readmission was 57.5 days (IQR 38-74). Infection was identified as the predominant cause of readmission, accounting for 23.1% (*n* = 3) of cases. The most frequently reported complications included respiratory failure (23.1%, *n* = 3), sepsis with septic shock (7.69%, *n* = 1), and neurological deterioration (15.4%, *n* = 2).

**Table 3:** Clinical characteristics and interventions of pediatric patients with HCT during the second admission to the PICU within the first 100 days post-transplant (*n* = 13).

Characteristic	Total number	Percentage
Days in PICU, median (IQR)	5 (3–6)	—
Days between transplant and re-admission to PICU, Median (IQR)	57.5 (38–74)	—
Cause of re-admission to PICU		
Infection	3	23.1
Neurological disorder	3	23.1
Bleeding	4	30.8
Other	3	23.1
Infection focus (*n* = 3)		
Bacteremia	2	66.7
Respiratory system	1	33.3
Isolated microorganism (*n* = 3)		
Klebsiella pneumoniae	1	33.3
Cytomegalovirus	1	33.3
Other (adenovirus)	1	33.3
Complications		
Ventilatory failure	3	23.1
Sepsis with septic shock	1	7.7
Neurological deterioration	2	15.4
Multiple organ failure	2	15.4
PRISM score		
Score 0–12	6	46.2
Score 13–19	2	15.4
Score >20	5	38.5
Requirement for mechanical ventilation	8	61.5
Type of ventilation		
Non-invasive mechanical ventilation (NIV)	2	15.4
Invasive mechanical ventilation (IMV)	6	46.2
Requirement for vasoactive support	5	38.5
Requirement for renal replacement therapy	2	15.4
Deceased patients	4	30.8
Cause of death during re-admission (*n* = 4)		
Multiple organ failure secondary to sepsis	2	15.4
Hypoxemic ventilatory failure	2	15.4

Among these patients, 46.2% (*n* = 6) had a PRISM III score ranging from 0 to 12. Mechanical Ventilation (MV) was required by 61.5% (*n* = 8) of patients, while 38.5% (*n* = 5) needed vasoactive support, and 15.4% (*n* = 2) required renal replacement therapy. There were 4 fatalities during this second admission. During the third admission, 2 out of 5 patients died.

In the comparative analysis of clinical characteristics based on patient outcomes (deceased = 27 vs. alive 42), the deceased group exhibited a significantly higher requirement for vasoactive support (81.4% vs. 23.8% *p* < 0.001), a higher proportion of multiple organ failure 37.0% vs. 0% (*p* < 0.000) and a higher requirement of renal replacement therapy 29.6% vs. 2.4% (*p* < 0.002). Additionally, a larger proportion of deceased patients had PRISM III scores exceeding 20 (59.3% vs. 30.9% *p* < 0.002). However, there were no statistically significant differences between the groups regarding the indication for transplantation, type of transplant, source of transplant cells, cause for PICU admission, as detailed in Table 4.

**Table 4:** Clinical characteristics of pediatric patients with HCT admitted to the PICU by final Status (*n* = 69).

	Deceased (*n* = 27) *n*, (%)	Alive (*n* = 42) n, (%)	Total (*n* = 69) *n*, (%)	*P*-value
Mechanical ventilation	19 (70.3)	22 (50.0)	41 (59.4)	*0.007*
Type of ventilation				*0.045*
NIV	4 (14.8)	9 (21.4)	13 (31.7)	
IMV	18 (66.5)	11 (26.2)	28 (68.3)	
Vasoactive support	22 (81.4)	10 (23.8)	32 (46.4)	*0.000*
Renal replacement therapy	8 (29.6)	1 (2.4)	9 (13.0)	*0.002*
Second admission to PICU	6 (22.2)	7 (16.6)	13 (18.8)	*0.650*
Days in PICU	12 (3–24)	4 (2–8)	5.50 (2.3–14)	*0.064*
Indications for transplant				*0.432*
Malignant disease/neoplasia	23 (85.2)	28 (66.6)	51 (73.9)	
Bone marrow failure syndrome	3 (11.1)	5 (11.9)	8 (11.6)	
Immunodeficiency	0 (0)	6 (14.3)	6 (8.7)	
Hemoglobinopathies	1 (3.70)	2 (4.8)	3 (4.4)	
Epidermolysis bullosa	0 (0)	1 (2.4)	1 (1.5)	
Type of transplant				*0.432*
Haploidentical related donor	23 (85.2)	27 (64.3)	50 (72.5)	
Identical related donor	2 (7.4)	7 (16.6)	9 (13.0)	
Autologous	2 (7.4)	6 (14.3)	8 (11.6)	
Unrelated donor	1 (3.7)	1 (2.4)	2 (2.9)	
Source of transplant cells				*0.828*
Peripheral blood	13 (48.1)	22 (52.4)	35 (50.7)	
Bone marrow	14 (51.9)	18 (42.9)	32 (46.4)	
Umbilical cord blood	1 (3.7)	1 (2.4)	2 (2.9)	
Cause of admission to PICU				
Infection	18 (66.6)	29 (69.0)	47 (68.1)	*0.573*
Bleeding	5 (18.5)	2 (4.8)	7 (10.1)	*0.080*
Neurological disorder	0 (0)	5 (11.9)	5 (7.3)	*0.055*
Graft-vs.-host disease	1 (3.7)	3 (7.1)	4 (5.8)	*0.513*
Electrolyte imbalances	6 (22.2)	3 (7.14)	9 (13.0)	*0.087*
Complications				
Sepsis with septic shock	13 (48.1)	12 (28.6)	25 (36.2)	*0.145*
Multiple organ failure	10 (37.0)	0 (0)	10 (14.5)	*0.000*
Neurological deterioration	1 (3.70)	7 (16.6)	8 (11.6)	*0.085*
Ventilatory failure	7 (25.9)	4 (9.5)	11 (15.9)	*0.089*
Other	2 (7.4)	4 (9.5)	6 (7.14)	*0.705*
PRISM III score				*0.002*
Score 0–12	6 (22.2)	26 (61.9)	32 (46.4)	
Score 13–19	4 (14.8)	5 (11.9)	9 (13.0)	
Score >20	16 (59.3)	13 (30.9)	28 (40.6)	

Italics value denotes that the p value was obtained by doing Chi-square test for categorical variables or he Mann Whitney U rank sum test for continuous variables.

**Figure 1:**
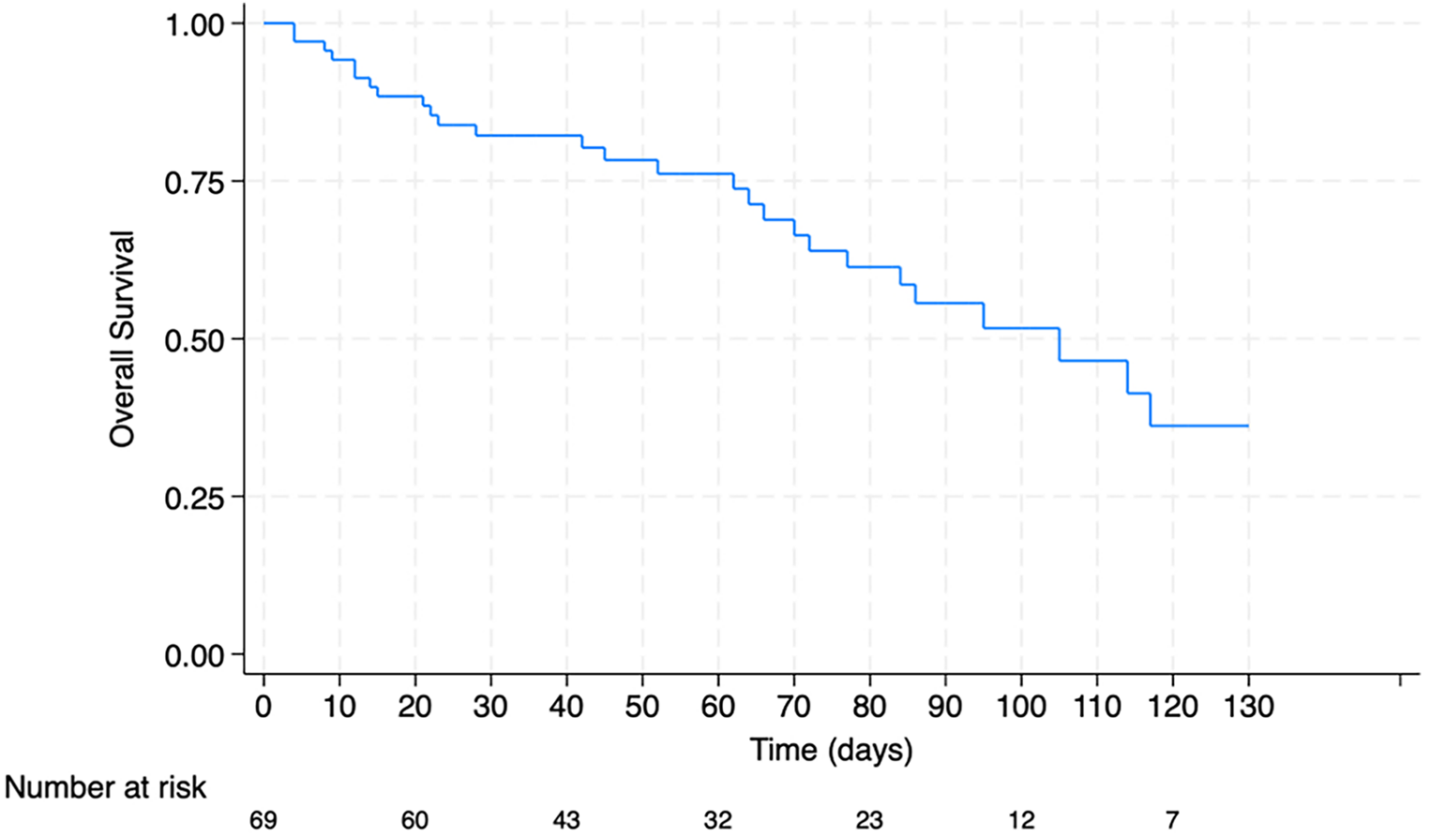
Overall survival rates of pediatric patients with HCT admitted to PICU.

Figure 1 shows a Kaplan Meier survival curve. Survival rate at 100 days after HCT was 51.6% among pediatric patients admitted to the PICU. Cox regression analysis showed that patients with PRISM III score above 20 points had a Hazard Ratio of 5.80 (CI95% 2.1–15.5) related to death.

## 4 Discussion

This study provides a comprehensive analysis of the clinical characteristics associated with mortality in pediatric patients who need PICU support within the first 100 days after their HCT at Fundación Valle del Lili, which is one of 8 hospitals in Colombia that offers pediatric HCT. Understanding the factors that contribute to mortality in these patients is crucial for improving outcomes, especially in LMICs.

In our study, 72.5% of patients underwent haploidentical transplants, which contrasts with global findings and the study conducted by Ozturk et al., reporting a significant higher proportion of transplants performed using matched related or unrelated donors (12). Although the adoption of allogeneic transplants is gradually increasing in our region, it remains in early stages (19, 20). This discrepancy reflects the ongoing challenge of finding fully compatible allogeneic donors and the lack of a comprehensive infrastructure to effectively identify and select unrelated donors through international registries (19). As a result, haploidentical transplants have emerged as a viable alternative in Latin America for patients who urgently require HCT but lack timely access to an HLA-compatible donor (21). The primary indications for HCTs in our patients were malignant diseases (73.9%), with acute lymphoblastic leukemia being the most frequently diagnosed condition, consistent with findings from other studies (11, 12, 19, 22). The incidence of PICU admissions among HCT patients in our cohort (21.8%) also aligns with internationally reported rates of 14.3% to 24% (9, 11, 12, 14, 15, 20).

Between January 2012 and December 2021, 316 HCTs were performed in pediatric patients at our institution, with 21.8% (*n* = 69) requiring PICU admission within the first 100 days post-transplant. The median age of patients admitted to the PICU was 8 years (IQR 4–13), with an equal sex distribution. Mortality was observed in 39.1% of these patients. Infectious etiology was the leading cause of admission accounting for 68.1% of our cases, paralleling the reported 68% by Torres et al. in Argentina (22, 23). Septic shock was the most prevalent complication, occurring in 36.2% of our patients during their initial PICU stay, mostly caused by Gram-negative bacteria. Although respiratory failure was noted in 15.9% of patients during their initial admission, it increased to 23.1% during their second admission, making it the most prevalent complication. These findings are consistent with other studies citing respiratory failure and septic shock as leading causes of PICU admission in post-transplant patients, both in Latin America and globally (11, 12, 16, 22, 23).

Among patients with infections, bacteria constituted the most frequently isolated microorganisms, accounting for 47.8% (*n* = 34) of cases. Enterobacteria, including *Klebsiella pneumoniae*, *Pseudomonas aeruginosa*, and *Escherichia coli,* are commonly associated with the neutropenic phase of illness and linked to more insidious infectious processes (11, 12, 24). In our study 47% of bacterial isolates were identified as carbapenemase producers. Therefore, patients required targeted antimicrobial therapy according to the resistance profile of the microorganism, which may extend recovery times and contribute to increased morbidity and adverse clinical outcomes (25, 26). Similar to a Polish multicenter study that included 308 post-HCT patients, which found that 78.6% of bacterial strains were multidrug-resistant (25). These findings highlight the critical need for vigilant monitoring and effective early interventions in this patient population, particularly for those with prior exposure to broad-spectrum antibiotics, catheter use, or those requiring MV (11, 12, 27). Bacteremia was the main infectious focus during both the initial and subsequent PICU admissions in our study.

The overall mortality rate was 39.1%, with 21 deaths occurring during the first PICU admission and an additional 4 and 2 deaths during the second and third admission, respectively. This is similar to research from Europe that reports in-PICU mortality rates ranging from 33% to 42.9% (11, 28), and is slightly higher than the 29% reported by Torres et al. in Argentina (22). However, data from the U.S. reports a lower mortality rate ranging from 16.2% to 22.1%, which can significantly increase in patients requiring MV (44.0%–60.4%) or renal replacement therapy (51.9%–56.1%) (15–17). In our study, mortality rates of patients with MV was 70.3%, while mortality rates in patients that required renal replacement therapy was 29.6%. This may be attributed to common challenges in the care of pediatric transplant patients across Latin America, including heterogeneity in access to intensive care and variability in the management of post-transplant complications. In LMIC, the difficulties in managing this population are often compounded by socioeconomic conditions and limited healthcare resources, which may further exacerbate poorer outcomes in these settings.

Kaplan-Meier survival analysis in our cohort revealed post-transplant survival rates for patients admitted to the PICU of 51.6% at 100 days. These results are comparable to Jensen et al.'s findings, and those by Schober et al. in Germany, which documented a survival rate of 60% at 3-months for patients admitted to the PICU due to HCT-related complications (28, 29). Notably, for now, survival data from Latin America remains scarce.

Several other key clinical factors were significantly associated with mortality in our patients, including a PRISM III score greater than 20 (*p* = 0.010), the need for mechanical ventilation (*p* = 0.034), renal replacement therapy (*p* = 0.003), vasoactive support (*p* < 0.001) and the presence of multiple organ failure (*p* = 0.003), aligning with existing literature (11, 12, 15, 17, 23, 30–33). In our study, 41.4% of the patients had a PRISM III score above 20, and 55.2% (*n* = 16) of these patients died. This observation is consistent with previous research highlighting the utility of PRISM III in assessing the severity of illness and predicting outcomes in critically ill pediatric patients more broadly but also in those with complications associated with HCT (9, 16, 27, 30, 32, 34, 35). In Latin America, Vásquez et al. demonstrated a significant association between PRISM III risk categories (low, moderate, and high), and mortality in children admitted to the ICU (*p* < 0.001), considering scores above 20 as indicative of a moderate risk for mortality (36). Similarly in our study, patients with PRISM III score above 20 had a 5.7 times higher chance of death during their PICU stay.

The timing of PICU admission post-transplant varied significantly between the initial and subsequent admissions. The first admission occurred at a median of 15 days post-transplant, indicating that most patients were admitted relatively soon after transplantation compared with international records (9). In contrast, the median time for the second admission extended to 17.5 days.

When evaluating therapeutic requirements during the first PICU stay, a staggering 59.4% of patients required invasive mechanical ventilation, and among those 70.3% did not survive. Vasopressor support was needed by 46.4% of patients, and 13.0% required renal replacement therapy. These data reflect the clinical severity of the patients’ conditions and the complexity of their care (15, 30, 31). Pillon et al. reported that patients undergoing multiple invasive interventions have a higher risk of adverse clinical outcomes, including prolonged PICU stay and increased mortality (11).

In contrast, the 13 patients who required a second PICU admission showed less severe conditions and better outcomes compared to their initial admission. The median length of stay during the second admission was equal (median stay of 5 days). Additionally, most of these patients 46.2% had a PRISM III score between 0 and 12, indicating a lower risk of mortality compared to their initial admission, and 38.5% had a score higher than 20 Tere was also a reduction in the need for invasive interventions, but mechanical ventilation remained necessary for 61.5% of these patients.

### 4.1 Strengths and limitations

This study is strengthened by a comprehensive 10-year study period, allowing an in-depth exploration of the clinical characteristics and outcomes associated with mortality in pediatric patients undergoing HCT. Conducted in Colombia, a setting with resource constraints, the study provides valuable insights that are directly applicable to similar contexts. By presenting data from environments lacking the advanced technologies and resources typically available in high-income countries, these findings significantly contribute to global literature on pediatric HCT.

Nonetheless, this study does have limitations. Its retrospective design inherently restricts the ability to establish causality and may introduce selection bias. The exclusion of patients treated at other institutions could further limit the generalizability of the results. Additionally, reliance on historical data and clinical records may have introduced information bias, potentially affecting the accuracy and completeness of the collected variables. Addressing these limitations in further research will be essential for enhancing the robustness and applicability of findings in diverse healthcare settings.

### 4.2 Conclusions

This study sheds lights on the clinical characteristics associated to increased early morbidity and mortality after HCT in pediatric patients admitted to a PICU in Colombia. Our findings revealed that a high PRISM III score, the need for mechanical ventilation, vasoactive support, and renal replacement therapy were significant predictors of mortality. Furthermore infection, especially those caused by multidrug-resistant bacteria, severely impacted our patients’ outcomes. Our findings emphasize the importance of early recognition and interventions of complications to improve survival rates for these patients especially in LMICs.

## Data Availability

The raw data supporting the conclusions of this article will be made available by the authors, without undue reservation.
